# Modifications of the Transcriptomic Profile of Autoreactive B Cells From Pemphigus Patients After Treatment With Rituximab or a Standard Corticosteroid Regimen

**DOI:** 10.3389/fimmu.2019.01794

**Published:** 2019-08-07

**Authors:** Vivien Hébert, Marie Petit, Maud Maho-Vaillant, Marie-Laure Golinski, Gaëtan Riou, Céline Derambure, Olivier Boyer, Pascal Joly, Sébastien Calbo

**Affiliations:** ^1^INSERM U1234, Normandie University, Rouen, France; ^2^Department of Dermatology, Rouen University Hospital, Normandie University, Rouen, France; ^3^INSERM U1245, Normandie University, Rouen, France

**Keywords:** pemphigus, rituximab, corticosteriods, autoreactive B cell, transcriptomic analysis

## Abstract

Pemphigus Vulgaris is an autoimmune disease of the skin and mucous membranes, which is due to the production of pathogenic autoantibodies targeting desmoglein (DSG) 1 and 3, which are adhesion proteins of the keratinocytes. Rituximab is an anti-CD20 mAb which induces a prolonged depletion of blood B cells. We recently showed that rituximab was more effective than a standard oral corticosteroid (CS) treatment, allowing 90% of patients to achieve complete remission (CR). Additionally, we showed that DSG-specific-B (DSG positive) cells were still detectable during the B cell recovery which follows the initial rituximab-induced B cell depletion, even in patients in CR. In order to characterize DSG positive B cells in patients in CR after rituximab or CS treatment relative to those detectable at baseline in patients with an active pemphigus, we studied the expression profile of 31 genes of interest related to inflammatory cytokines, TNF receptors and activation markers. Using quantitative Polymerase Chain Reaction performed on one cell with a microfluidic technique, we found that patients' autoreactive B cells collected at baseline had a significantly higher expression of genes encoding for IL-1β, IL-23p19, and IL-12p35 pro-inflammatory cytokines and the IRF5 transcription factor, than non-autoreactive B cells. Surprisingly, the gene expression profile of DSG positive B cells collected after rituximab treatment in patients in CR was close to that of DSG positive B cells at baseline in patients with active pemphigus, except for the IL-1β and the CD27 memory marker genes, which were under-expressed after rituximab compared to baseline. Conversely, we observed a decreased expression of genes encoding for IL-1β and IL-23p19 in patients treated with CS relative to baseline. This study showed that: (i) DSG positive autoreactive B cells have a different gene expression profile than non-autoreactive B cells; (ii) rituximab and CS have different effects on the genes' expression in autoreactive DSG positive B cells from pemphigus patients.

## Introduction

Pemphigus is a rare life-threatening autoimmune blistering disease involving the skin and mucosa, leading to erosions and major weight loss, which severely impair patients' quality of life (1–5). It is characterized by the production of pathogenic autoantibodies directed against two desmosomal proteins involved in keratinocyte adhesion: desmoglein 1 (DSG1) and desmoglein 3 (DSG3) (1, 6). DSG3 is mainly found in deep skin epidermal layers and mucosa, while DSG1 is present throughout the epidermis. Anti-DSG3 antibodies (Abs) are observed in pemphigus vulgaris (PV) with mucosal involvement, and anti-DSG1 Abs are observed in patients with pemphigus foliaceus (PF) and those with PV and skin involvement. Binding of anti-DSG Abs to their target antigen results in a loss of keratinocyte adhesion, leading to the so called “acantholysis” phenomenon, which results in the formation of skin and mucosal blisters (7–9).

Until recently, high-dose of oral corticosteroids (CS) sometimes associated with immunosuppressive drugs (azathioprine, mycophenolate mofetil) were the mainstay of pemphigus treatment (10). A recent large randomized controlled study (RCT) from our group demonstrated the interest of the first line use of rituximab in pemphigus (11). A large difference was evidenced in the primary outcome: 89% of patients in the rituximab + short term CS arm were in complete remission off therapy after 2 years, compared with 34% of patients treated with prednisone alone (*p* < 0001).

Rituximab is a chimeric murine-human monoclonal antibody that binds to the CD20 antigen of B-lymphocytes (anti-CD20 mAb), which is expressed by pre-B-cell and pre-plasma cells. A stop in the renewal of the plasma cell pool is thought to be one of the main mechanisms of the short-term action of rituximab (12, 13). However, the long-term clinical course of pemphigus patients treated with rituximab has not been extensively assessed yet. In particular, apart from a reversal of the naïve/memory B-cell *ratio*, the long-term evolution of reappearing DSG1– and DSG3–specific B cells after rituximab treatment remains largely unknown (14). Furthermore, the effects of oral CS on DSG specific-B cells in pemphigus patients has not been studied yet.

In addition to producing antibodies, B cells secrete multiple cytokines with pro- or anti-inflammatory functions, which can either enhance or downregulate the immune response. Since understanding the long-term modifications of the DSG-specific autoimmune B cell response after rituximab treatment might improve the way we use this drug, we longitudinally analyzed the transcriptomic and phenotypic profiles of one-cell sorted autoreactive DSG-specific B cells from patients with a newly diagnosed pemphigus (during the active phase of disease) and after treatment with rituximab or a standard CS regimen, and compared these results with those observed from non-autoreactive B cells.

## Results

### Baseline Analysis

**Figure 1:**
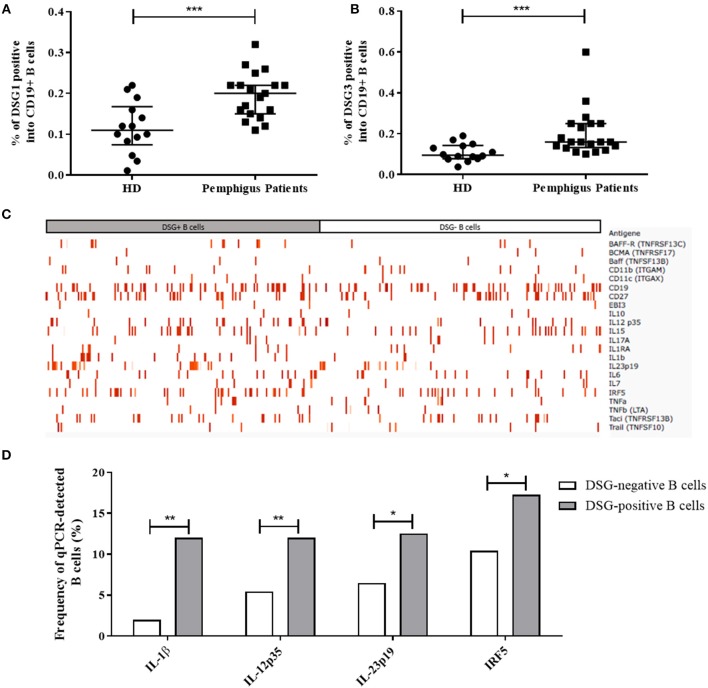
**(A,B)** Percentage of autoreactive DSG1-positive (CD19+DSG1+) B cells **(A)** and DSG3-positive B cells (CD19+DSG3+) **(B)** among total B cells (CD19+) collected in 14 healthy donors and 20 pemphigus patients at baseline. Median with interquartile range were compared using the Mann-Whitney *t*-test. **(C)** Intensity of gene expression in one-cell sorted DSG-positive B cells (in gray *n* = 191 cells) and non-autoreactive DSG-negative B cells (in white, *n* = 201 cells) from pemphigus patients. The color intensity in each sorted cell depended on the ΔCT value relative to GAPDH house-keeping gene expression. Non-detected genes in one-cell qPCR were excluded from the map. **(D)** Comparison of the baseline frequencies of cells expressing the IL-1β, IL-12p35, IL-23p19 genes, and the transcription factor IRF5 gene between DSG-positive autoreactive B cells (gray columns, *n* = 191 cells) and non-autoreactive DSG-negative B cells (white columns, *n* = 201 cells) from pemphigus patients. Frequencies were compared using the Fisher's exact test.

Using quantitative polymerase chain reaction (qPCR), we analyzed the single cell mRNA expression of 33 genes encoding for membrane markers, cytokine receptors or transcription factors (Table SI) which are known to be expressed in B cells from 20 patients. Among them, nine pemphigus patients were treated by rituximab and assessed at baseline and at Month 36 (18 months after the last rituximab infusion); all of them were in complete remission at M36. Eleven pemphigus patients were treated by oral corticosteroids and assessed at baseline and Month 12, after tapering of CS doses; five of these 11 patients were in clinical complete remission at Month 12. The 20 patients had a pemphigus vulgaris with either isolated mucosal involvement or skin and mucosal involvement. Clinical, biological, and immunologic characteristics of patients are presented in Tables 1, 2. Finally, we performed a transcriptomic analysis in seven out of 14 healthy donors (HD) who were studied with phenotypic analyses. Indeed, DSG1-positive (Figure 1A) and DSG3-positive (Figure 1B) B cells were detectable in HD, although at a lower frequency than in the 20 patients with active pemphigus (DSG1 positive 0.11 vs. 0.20%, *p* < 0.001; DSG3 positive 0.10% vs. 0.20%, *p* < 0.001).

**Table 1:** Clinical and biological characteristics of patients from the rituximab group.

**Age**	**Treatment**	**Type of pemphigus**	**Severity**	**ELISA anti-DSG1 Ab value at D0**	**ELISA anti-DSG3 Ab value at D0**	**Clinical status at M36**	**ELISA anti-DSG1 Ab value at M36**	**ELISA anti-DSG3 Ab value at M36**
51	RTX	Mucosal	Moderate	30	750	Complete remission	<20	80
45	RTX	Mucosal	Severe	<20	360	Complete remission	<20	<20
63	RTX	Mucosal	Severe	<20	2,050	Complete remission	<20	111
28	RTX	PV skin + mucosa	Severe	330	120	Complete remission	<20	555
76	RTX	Mucosal	Severe	<20	530	Complete remission	<20	157
80	RTX	PV skin + mucosa	Severe	410	1,805	Complete remission	<20	<20
52	RTX	PV skin + mucosa	Severe	260	4,000	Complete remission	<20	<20
64	RTX	PV skin + mucosa	Severe	200	131	Complete remission	<20	<20
48	RTX	PV skin + mucosa	Severe	356	176	Complete remission	<20	<20

**Table 2:** Clinical and biological characteristics of patients from the standard corticosteroids (CS) group.

**Age**	**Type of pemphigus**	**Severity**	**ELISA anti-DSG1 Ab value at D0**	**ELISA anti-DSG3 Ab value at D0**	**Clinical status at M12**	**ELISA anti-DSG1 Ab value at M12**	**ELISA anti-DSG3 Ab value at M12**
76	PV skin + mucosal	Severe	107	2,400	Complete remission	<20	<20
54	PV skin + mucosal	Severe	1,960	179	Complete remission	<20	<20
66	PV skin + Mucosal	Severe	27	3,350	Complete remission	<20	126
48	PV skin + mucosa	Severe	176	760	Complete remission	<20	<20
33	PV skin + mucosa	Severe	60	143	complete remission	<20	<20
79	PV skin + mucosa	Severe	1,110	553	Incomplete remission	<20	<20
45	PV skin + mucosa	Severe	156	5,217	Incomplete remission	30	1040
61	PV skin + mucosa	Severe	170	1,750	Incomplete remission	<20	550
71	PV skin + mucosa	Severe	665	225	Incomplete remission	77	<20
58	PV skin + mucosa	Severe	1,070	1,065	Incomplete remission	<20	<20
58	PV skin + mucosa	Severe	214	1,979	Incomplete remission	<20	128

We first compared the baseline gene expression between non-autoreactive DSG-negative B cells (201 single cells) and autoreactive DSG-positive B cells (191 single cells) from pemphigus patients. We observed that the mRNA of 11 genes (IL-2, IL-5, IL-9, IL-12p40, IL-13, IL-17F, IL-21, IL-27p28, IFNγ, TGF β2, and APRIL) was not detected at baseline neither in DSG-positive, nor in DSG negative B cells from pemphigus patients, whereas all housekeeping genes were detected. Therefore, these latter genes were not further analyzed. The 22 remaining genes detected were used to realize a heat map representing the ΔCT expression of these genes between autoreactive and non-autoreactive B cells at baseline (Figure 1C).

At baseline, autoreactive DSG-positive and non-autoreactive DSG-negative sorted B cells showed distinct mRNA expression profiles for the three pro-inflammatory cytokine genes IL-1β, IL-12p35, IL-23p19, and for the transcription factor IRF5 gene which were found to be overexpressed by the DSG-positive relative to the DSG-negative B cell populations (Figure 1D), whereas no difference of expression of these four genes was evidenced between DSG-positive (107 single cells) and DSG-negative (69 single cells) B cell populations from HD (data not shown).

### Longitudinal Analyses in Patients Treated With Rituximab

**Figure 2:**
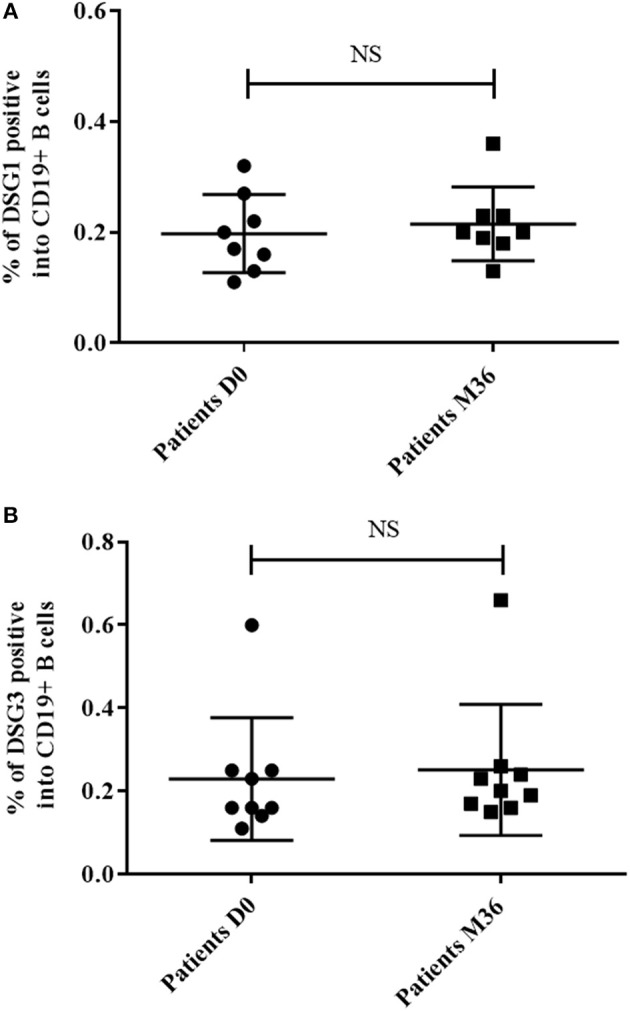
Percentage of autoreactive DSG1-positive B cells (CD19+DSG1+) **(A)** and DSG3-positive B cells (CD19+DSG3+) **(B)** among total B cells (CD19+) collected in pemphigus patients at baseline and at Month 36 after rituximab treatment. Means ± standard deviations were compared using the Wilcoxon paired test.

We performed a longitudinal analysis by flow cytometry in the nine rituximab-treated pemphigus patients previously analyzed by qPCR. Frequency of DSG1-positive and DSG3-positive autoreactive B cells did not vary significantly between baseline and Month 36 when patients were in complete remission (DSG1 positive: 0.20% vs. 0.22%, *p* = 0.67; DSG3 positive 0.23% vs. 0.25, *p* = 0.61; Figures 2A,B).

**Figure 3:**
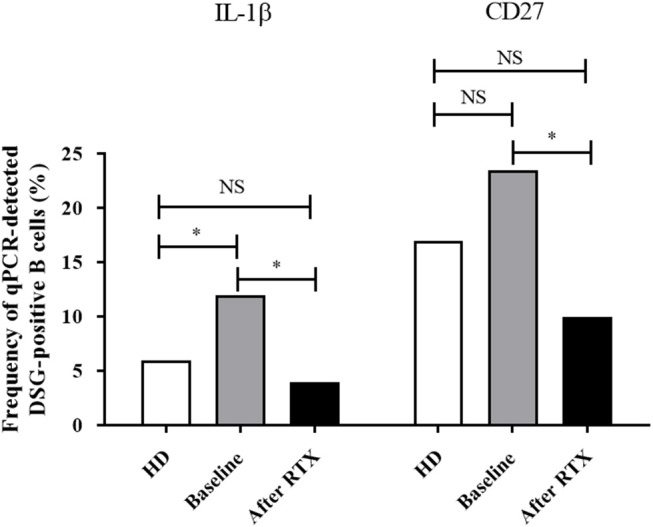
Frequency of cells with detectable mRNA encoding for IL-1β and CD27 in one-cell sorted desmoglein-positive autoreactive B cells from healthy donors (white columns *n* = 107 cells) compared to pemphigus patients before treatment (gray columns, *n* = 191 cells) or after rituximab treatment (black columns, *n* = 115 cells). Frequencies were compared using the Fisher's exact test.

We then analyzed the expression of selected genes in 306 single DSG-positive B cells from the nine rituximab-treated pemphigus patients, including 191 sorted-cells from blood samples collected at baseline and 115 sorted-cells from samples collected at Month 36 after RTX treatment. One hundred and seven single DSG-positive B cells from the seven healthy individuals were used as controls (Figure 3).

Interestingly, the expression of the IL-1β gene in DSG-positive B cells, which was higher at baseline in pemphigus patients than in healthy individuals (12 vs. 6%, *p* = 0.048), returned to normal values after rituximab treatment (12 vs. 4%, *p* = 0.039). We also observed that, whereas the frequency of expression of the CD27 memory marker gene in desmoglein-positive B cells collected from pemphigus patients at baseline was close to that observed in healthy individuals (23 vs. 17%, *p* = 0.24), the CD27 gene expression decreased from baseline to Month 36 in pemphigus patients treated with rituximab (23 vs. 10%, *p* = 0.03).

Surprisingly, we did not observe any statistically significant variation in the frequency of the other genes studied between samples collected from pemphigus patients at baseline and after rituximab treatment.

**Figure 4:**
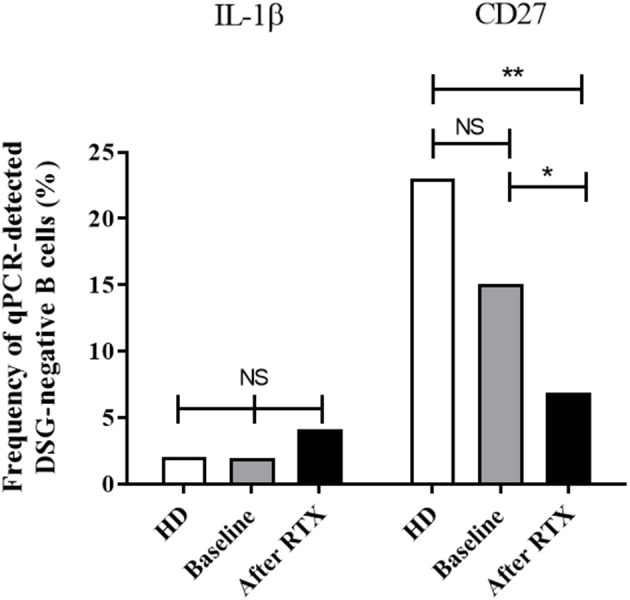
Frequency of cells with detectable mRNA encoding for the IL-1β and CD27 genes in one-cell sorted desmoglein-negative B cells from healthy donors (white columns, *n* = 69 cells) and pemphigus patients at baseline (gray columns, *n* = 201 cells) and after rituximab treatment (black columns, *n* = 106 cells). Frequencies were compared using the Fisher's exact test.

In non-autoreactive desmoglein-negative B cells, rituximab treatment did not induce modification of gene expression for the pro-inflammatory cytokine IL-1β (2 vs. 4.1%, *p* = 0.73) whereas it induced a decreased frequency of expression of the CD27 memory marker gene (15.1 vs. 6.9%, *p* = 0.04; Figure 4).

### Longitudinal Analyses in Patients Treated With Corticosteroids Alone

We performed a longitudinal flow cytometry analysis in the same 11 corticosteroids-treated pemphigus patients previously analyzed by qPCR. In these 11 CS-treated patients, 311 single DSG-positive cells were isolated including 191 sorted-cells from samples collected at baseline and 120 sorted-cells from samples collected at Month 12. This Month 12 evaluation was chosen because patients still received low doses of prednisone at this time point.

**Figure 5:**
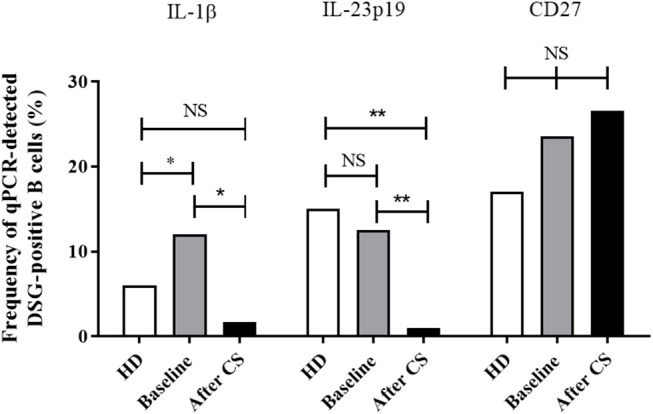
Frequency of cells with detectable mRNA encoding for IL-1β, IL-23p19, and CD27 in one-cell sorted desmoglein-positive from healthy donors (white columns, *n* = 107 cells) compared to pemphigus patients before treatment before (gray columns, *n* = 191 cells) and after CS treatment (black columns, *n* = 120 cells). Frequencies were compared using the Fisher's exact test.

After treatment with systemic corticosteroids (Figure 5), IL-1β gene was downregulated (12–1.7%, *p* = 0.003) allowing to return to values close to those observed in HD. Although the baseline gene expression of the pro-inflammatory cytokine IL-23p19 in desmoglein-positive B cells from patients was close to that detected in healthy individuals (12.5 vs. 15%, *p* = 0.59), we observed a downregulation of the gene after corticosteroids treatment (12.5 vs. 1.2%, *p* < 0.005). We did not observe significant variation in the expression of the other genes studied, including the CD27 memory marker gene (baseline: 23.5% vs. after CS treatment 26.6%, *p* = 0.48).

**Figure 6:**
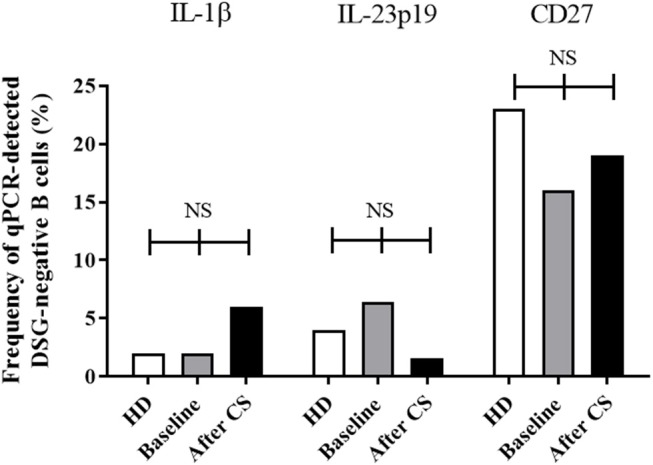
Frequency of cells with detectable mRNA encoding for IL-1β, IL-23p19, and CD27 in one-cell sorted desmoglein-negative B cells from healthy donors (white columns, *n* = 69 cells), and pemphigus patients at baseline before treatment (gray columns, *n* = 201 cells) or after corticosteroids treatment (black columns, *n* = 123 cells). Frequencies were compared using the Fisher's exact test.

In non-autoreactive desmoglein-negative B cells (Figure 6), corticosteroids treatment induced non-significant variations of the IL-23p19, IL-1β, and CD27 genes, as other genes from the panel studied.

### Assessment of the CD27 Memory Marker in Autoreactive B Cells After Rituximab

**Figure 7:**
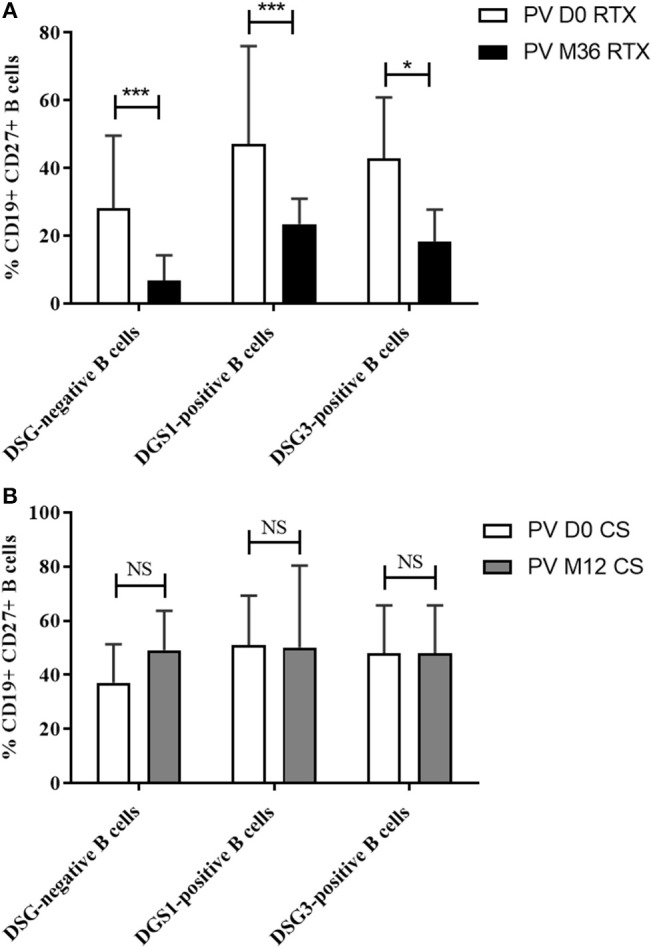
**(A)** Percentage of memory B cells among non-desmoglein specific, desmoglein 1-specific (DSG1+), and desmogelin three-specific (DSG3+) B cells before (white columns) and after treatment with rituximab (black columns). **(B)** Percentage of memory B cells among non-desmoglein specific, desmoglein 1-specific (DSG1+), and desmogelin 3-specific (DSG3+) B cells before (white columns) and after treatment with corticosteroids (gray columns). Means ± standard deviations were compared using the Wilcoxon paired test.

To extend the results of our transciptomic analysis, we studied the evolution of the CD27 expression on the cell surface of DSG-positive and DSG-negative B cells during treatment with rituximab or corticosteroids. According to the findings from transcriptomic analyses, we observed a significant decrease in the frequency of memory CD19+CD27+ autoreactive DSG-positive and DSG-negative B cells after rituximab treatment (Figure 7A), that were not observed in patients treated with CS alone without rituximab (Figure 7B).

### ELISA Assays

In order to evaluate the consequences of the decreased IL-1β gene expression in DSG-positive B cells from pemphigus patients whether they were treated with corticosteroids or rituximab, we performed longitudinal dosages of serum IL-1β using ELISA assay.

**Figure 8:**
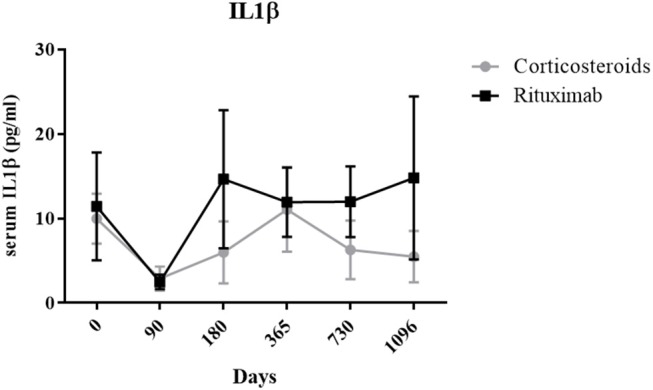
Longitudinal analysis of mean serum IL-1β level in rituximab (black) and corticosteroids-treated (gray) patients. Means ± standard error of the mean were compared with baseline level in each group (RTX and CS) using one-way analysis of variance followed by Bonferroni *post-hoc* test.

We observed a non-significant decrease of serum IL-1β ELISA values in pemphigus patients during the first 3 months of treatment, that was secondarily followed by a re increase of serum levels to baseline values when corticosteroids doses were tapered during patients' follow-up (Figure 8).

Finally, the IL-23p19 cytokine, whose gene expression was downregulated after CS treatment in autoreactive B cells, was also analyzed by ELISA. No significant variation in the IL-23p19 serum level was detected in pemphigus patients whether they were treated with rituximab or CS alone (data not shown).

## Discussion

In this study, we assessed the transcriptomic profiles of 33 genes of potential interest in pemphigus on one-cell sorted autoreactive B cells collected from pemphigus patients at different stages of their disease, in particular during the acute phase of the disease, and after two treatments (systemic corticosteroids alone or associated with rituximab) which were compared in a randomized controlled trial (11).

This methodology has several major interests. First, it allowed to study *ex vivo* autoreactive B-lymphocytes, since pemphigus is one of the rare autoimmune diseases in humans in which autoantibodies directed against DSG are pathogenic (15). Second, auto-reactive DSG positive B cells are the pre-stage of antibody-secreting plasma cells, which cannot be easily studied *ex vivo* in humans. Third, the one-cell sorting assays are likely relevant since a very low number of circulating autoreactive B cells in the peripheral blood are enough to trigger the disease, as we showed in the baseline analyses. To the best of our knowledge, this single-cell transcriptome analysis of autoreactive cells is the first one performed in patients with an auto immune disorder.

We first evidenced that the transcriptomic profiles of autoreactive and non-autoreactive B cells collected during the active phase of disease were different. Four out of the 31 genes studied (IL-1β, IL-12p35, IL-23p19, IRF5) were overexpressed in autoreactive relative to non-autoreactive B cells. The most surprising finding in this study was the observation that in addition to the fact that DSG-positive B cells are still detectable after rituximab treatment, arguing against a simple deletion mechanism, the gene expression profile of DSG-positive autoreactive B cells collected after rituximab treatment in patients in complete remission was actually pretty close to that of DSG-positive B cells collected at baseline in patients with active pemphigus, except for the IL-1β and the CD27 genes, which were downregulated after rituximab compared to baseline.

The downregulation of the CD27 memory marker gene observed in transcriptomic analysis was confirmed by flow cytometry, which showed a lower expression of the CD27 protein at the cell surface of autoreactive B cells after rituximab as well as in non-autoreactive B cells. Thus, the decreased expression of the CD27 gene after rituximab was not specific for DSG-positive B cells. However, the decreased expression of the CD27 marker reflects the blockage of B cell maturation, leading to a prolonged repopulation with naïve B cells, and a delayed reappearance of memory B cells. It has been suggested that autoantibodies are produced by a transitory subpopulation of autoreactive plasmablasts which are continuously refund from autoreactive switched memory B cells (CD20+IgG+CD27+) (13, 16). Thus, changes in CD27 expression which correlates with the disappearance of circulating DSG-positive IgG(+) B lymphocytes are likely responsible for the decreased production of anti-DSG autoantibodies, that contributes to the long lasting clinical remission of the disease after rituximab treatment (14). Another argument which supports this hypothesis is the fact that by using an ELISPOT assay, we showed that DSG3+CD27+ B cells but not DSG3+CD27− B cells are able to differentiate into antibody secreting cells after 72 h of stimulation by IL-2 and R848, a TLR7 and eight ligands (still unpublished data). This mechanism was specifically related to rituximab, since it was not observed in patients treated with CS alone, whether they were in complete or incomplete remission.

Conversely, changes in CD27 do not influence the production of alloreactive antibodies which are produced by long-lived plasma cells which do not express the CD20 marker, explaining the absence of modification of total serum IgG levels in after rituximab in most patients.

We also observed that the expression of the IL-1β gene in DSG specific B cells, which was higher in baseline samples from pemphigus patients than in healthy individuals, and returned to normal values after rituximab or oral CS treatment, whereas no modification was observed in non-autoreactive desmoglein-negative B cells. This observation might suggest the potential role of IL-1β in the pathogenicity of pemphigus, since the downregulation observed in autoreactive B cells paralleled disease remission, whether patients were treated with rituximab or oral CS. Indeed, IL-1β is a pro-inflammatory cytokine mainly produced by hematopoietic cells such as blood monocytes, tissue macrophages and skin dendritic cells in response to TLR ligand, activated complement components or other cytokines such as TNF-α and IL-1β itself (17, 18). In our work, we observed a slight decrease of serum IL-1β during the first 3 months of treatment in both CS- and RTX-treated groups, which was likely related to the effect of high doses of CS. It was then followed by a re-increase of serum levels to baseline values when corticosteroids doses were tapered. The role of IL-1β has previously been suggested by Feliciani et al. who demonstrated that an IL-1β KO mouse model was less susceptible to passive transfer of IgG isolated from the serum of a PV patient. Moreover, Increased concentrations of IL-1α and IL-1β were detected in the serum of untreated PV patients with active disease relative to healthy controls. Additionally, the *in vivo* and *in vitro* production of IL-1α and IL-1β decreased in patients in clinical remission after treatment with IVIG. Finally, IL-1β promotes Th17 polarization, which correlates with disease activity (19–24).

Since the frequency of autoreactive B cells in pemphigus patients was surprisingly only doubled compared to healthy donors, it is likely that the different gene expression profiles of IL-1β and CD27 play a major role in the onset of pemphigus by promoting the emergence/appearance of DSG + CD27+ IgG+ B memory cells, which were only detected in pemphigus patients but not in healthy individuals.

Despite the fact that we observed a difference in the baseline expression of the IL-12p35 between DSG-positive and DSG-negative B cells, we did not observe significant modification in RNA expression in patients in complete remission after rituximab, nor variation in the serum concentration of IL-12p35 during patients' follow-up, which does not support a major role for IL-12p35 in the healing of RTX-treated patients.

We did not analyse the serum concentration of cytokines relevant for B cell activation such as BAFF, APRIL, or IL-6, because we did not observe any modification of the gene expression of these cytokines by autoreactive DSG-positive B cells in our transcriptomic analyses.

Overall, this study showed that self-reactive and non-self-reactive B cell populations from pemphigus patients do not express the same genes during the acute phase of the disease, in particular genes encoding for IL-1β, IL-12p35, IL-23p19, and IRF5. Additionally, DSG-positive autoreactive B cells can still be observed in patients in complete remission, even in those treated with rituximab, when blood B-lymphocytes reappeared after the initial rituximab-induced depletion. Surprisingly, the gene expression of autoreactive DSG-positive B cells in remitted patients was close to that observed at baseline, except for the CD27 gene which was downregulated in patients treated with rituximab, and the IL-1β gene, which was down regulated in both treatment groups, likely related to the effect of oral CS.

## Methods

### Clinical Study

In the randomized controlled trial, 90 newly-diagnosed pemphigus patients were randomly assigned to receive a standard regimen of CS vs. rituximab associated with a short-term CS regimen. Patients in the rituximab group were treated with the autoimmune regimen (two infusions of 1,000 mg of rituximab at day 0 and day 14) and a maintenance treatment corresponding to two infusions of 500 mg at Month 12 and Month 18. They also received an initial medium dose of prednisone, 0.5–1 mg/kg/day, depending on initial pemphigus severity (moderate vs. severe), which was rapidly tapered over 3–6 months. Patients assigned in the standard oral CS group were given a higher initial dose of prednisone, 1–1.5 mg/kg/day, with a progressive tapering over 12–18 months, depending on initial pemphigus severity. Patients who participated in the Ritux 3 clinical trial were included in the present study (ClinicalTrials.gov number, NCT00784589).

### Patients

Blood samples from rituximab-treated patients were analyzed at baseline before treatment (Day 0) and after 36 months of follow-up, since we had to wait for the recovery of blood B-lymphocytes after the repeated infusions of rituximab at Day 1 and Month 12 and Month 18. Blood samples from CS-treated patients were analyzed before treatment and at Month 12 at which time patients in the standard CS group were still treated with oral CS.

### Serum Auto-Antibody Titers and Cytokine Levels

Titers of IgG antibodies against DSG1 and three were measured by a DSG enzyme-linked immunosorbent assay test (ELISA) with 1:100 diluted serums (EUROIMMUN, Germany, Lübeck). Serum levels of IL-1β and Il-23p19 were quantified by ELISA (RD and ebioscience, respectively), according to the manufacturer's protocol.

### Phenotyping of Desmoglein-Specific B Cells

In order to analyse DSG1 and DSG3 specific B cells, B cells were isolated using Dynabeads Untouched Human B-cells kit (Life Technologies) according to the manufacturer's instruction. Then, purified B cells were incubated for 30 min at 4°C with histidine-tagged recombinant DSG1 or DSG3 (30 ng/μl). After washing, B cells were stained with anti-human IgG antibodies (BD Biosciences). Cells were then incubated with Fc Block (eBioscience), LIVE/DEAD Fixable Aqua Dead Cell Stain (Life Technologies), anti-human antibodies directed against CD19, CD27, IgM (BD Biosciences). Anti-histidine coupled with phycoerythrin (R&D Systems) was used to identify desmoglein-specific B cells.

### One-Cell Sorting and Pre-amplification

DSG-positive single B cells were sorted using FACS ARIA III into 96-well plates containing 10μL Platinum Taq polymerase and SuperScript III reverse transcriptase (Invitrogen), a mixture of Taqman primer-probes at 0.2× concentration specific for the transcripts of interest (Table SI) and CellsDirect qRT-PCR buffer (Invitrogen). Immediately after cell sorting, samples were centrifuged, incubated at 55°C for 10 min, and subjected to 30 cycles of PCR (50°C 15 min then 95°C for 15 s for the reverse transcription, followed by 30 cycles of 95°C 15 s, and 60°C 4 min for amplification). Subsequent pre-amplified single-cell cDNA was stored at −20°C until analysis.

### Real-Time qPCR

After ¼ dilution in TE buffer, each cDNA sample was then separated into 48 separate reactions for further qPCR using the BioMark 48.48 dynamic array nanofluidic chip (Fluidigm, Inc.). Briefly, following hydraulic chip priming, 48 pre-amplified cDNA samples were mixed with a mild detergent loading solution to allow capillary flow. Samples were then added to a 48.48 nanofluidic chip (Fluidigm, Inc.) along with 38 individual Taqman primer-probe mixtures listed in Table SI (Applied Biosystems) specific for individual transcripts of interest, allowing a combination of each sample to mix with each probe in every possible combination (a total of 2,304 reactions). The chip was then thermocycled through 40 cycles and fluorescence in the FAM channel was detected using a CCD camera placed above the chip, normalized by ROX (6-carboxy-X-rhodamine) intensity. A well containing 100 CD19+ cells and a well without cell were used as positive and negative controls, respectively. To limit potentially biased measurement, cells with <2 expressed genes among the five control genes (HPRT1, B2M, GUSB, TUBB, and GAPDH) were excluded from the analysis. Data were analyzed using Real Time PCR Analysis software with or without normalization of the Ct value for each gene using GAPDH as calibrator gene. We considered that sorted cells expressed a gene if the Ct (threshold of detection) value was <40 and if the detection curve was a sigmoid. Eleven genes were found to be unexpressed by single B cells including IL-2, IL-5, IL-9, IL-12p40, IL-13, IL-17F, IL-21, IL-27p28, IFNγ, TGFβ2, and APRIL. This absence of detection was due to a too low RNA amount since positive control wells containing 100 CD19+ cells showed detectable expression levels of all tested cytokine genes. We compared the frequency of cytokine gene expressing B cells using qPCR between treatment groups.

### ΔCt Analysis

The ΔCt analysis allows to determine the intensity of the gene of interest expression in cells by comparing its threshold of detection (Ct) with the Ct of the housekeeping gene.

### Statistical Analysis

Statistical comparison of the frequency of gene expressing cells was performed with Fisher's exact test. Statistical comparison of percentages of autoreactive B cells in flow cytometry were calculated using the Mann-Whitney *t*-test to compare healthy donors and pemphigus patients; and using the Wilcoxon *t*-test to compare patients before and after treatment. All analyses were performed using the GraphPad Prism Software. A *p*-value ≤ 0.05 was considered as significant. The *, **, *** refer to statistical values explained in the legends.

## Data Availability

The raw data supporting the conclusions of this manuscript will be made available by the authors, without undue reservation, to any qualified researcher.

## Author Contributions

VH: transcriptomic, cytometric acquisitions, analyses, ELISA assays, and writer. MP: transcriptomic acquisitions. MM-V: transcriptomic, cytometric acquisitions, and ELISA assays. M-LG: cytometric acquisitions. GR: cytometric analyses. CD: transcriptomic analyses. OB: drafting and revision. SC and PJ: transcriptomic analyses, cytometric analyses, drafting, and revision.

### Conflict of Interest Statement

The authors declare that the research was conducted in the absence of any commercial or financial relationships that could be construed as a potential conflict of interest.
