# Supplementary material for: Modifications of the Transcriptomic Profile of Autoreactive B Cells From Pemphigus Patients After Treatment With Rituximab or a Standard Corticosteroid Regimen
Source: Front Immunol. 2019 Aug 7;10:1794. doi: 10.3389/fimmu.2019.01794 (PMC6693356; doi:10.3389/fimmu.2019.01794)
Supplement: Supplementary file 1 [file Table_1.docx]

| **Gene Name** | **Assay ID** |
| --- | --- |
| **Cytokine Genes** |  |
| IL-1β | Hs01555410_m1 |
| IL-1RA | Hs00893626_m1 |
| IL-2 | Hs00174114_m1 |
| IL-5 | Hs01548712_g1 |
| IL-6 | Hs00985639_m1 |
| IL-7 | Hs00174202_m1 |
| IL-9 | Hs00914237_m1 |
| IL-10 | Hs00961622_m1 |
| IL-12A | Hs01073447_m1 |
| IL-12B | Hs01011518_m1 |
| IL-13 | Hs00174379_m1 |
| IL-15 | Hs01003716_m1 |
| IL-17A | Hs00174383_m1 |
| IL-17F | Hs00369400_m1 |
| IFNγ | Hs00989291_m1 |
| TNFα | Hs01113624_g1 |
| LTA | Hs04188773_g1 |
| TNFSF10 | Hs00921974_m1 |
| IL-21 | Hs00222327_m1 |
| TGFβ2 | Hs00234244_m1 |
| TNFSF13B | Hs00198106_m1 |
| TNFSF13 | Hs00601664_g1 |
| EBI3 | Hs01057148_m1 |
| IL-27 | Hs00377366_m1 |
| IL-23A | Hs00900828_g1 |
| Taci (TNFRSF13B) | Hs00963364_m1 |
| BCMA (TNFRSF17) | Hs03045080_g1 |
| BAFF-R (TNFRSF13C) | Hs00606874_g1 |
| CD11b (ITGAM) | Hs00355885_m1 |
| CD11c (ITGAX) | Hs00174217_m1 |
| CD27 | Hs00386811-m1 |
| CD19 | Hs00174333-m1 |
| IRF5 | Hs00158114_m1 |
| **Housekeeping Genes** |  |
| HPRT1 | Hs02800695_m1 |
| B2M | Hs00984230_m1 |
| GUSB | Hs00939627_m1 |
| TUBB | Hs00742828_s1 |
| GAPDH | Hs02758991_g1 |

**Table SI:** Taqman primers used in High Throughput qPCR Biomark analysis.
